# News and Events of the Week

**Published:** 1910-03-12

**Authors:** 


### News and Events of the Week.

Professor Sir J. Thomson has been nominated to represent Cambridge University at the celebration, in October 1910, of the centenary of the University of Berlin.

The Congress and Health Exhibition of the Royal Sanitary Institute will be opened in the Royal Pavilion, Corn Exchange, Brighton, on September 5, and will remain open until September 14 next.

At the meeting of the Society of Medical Officers of Health, to be held at 1 Upper Montagu Street, Russell Square, W.C., on Friday, April 8, 1910, a Paper will be read on "The Majority Report of the Royal Commission on the Poor Law" by C. S. Loch, Esq., B.A., D.C.L., LL.D.

The thirty-ninth congress of the German Society for Surgery will be held in Berlin, beginning Wednesday morning, March 30, 1910, and lasting until Saturday, April 2, 1910, in the Langenbeckhaus Ziegelstrasse 10-11, under the chairmanship of Professor S. Bier. Membership cards and information may be obtained from Herr Melzer, Langenbeckhaus, Berlin.

At the annual general meeting of the Newsvendors' Benevolent and Provident Institution, on February 15 last, under the chairmanship of Colonel the Hon. H. L. W. Lawson, M.P., the committee gave notice that Charles Skeats had been elected to be the recipient of The Hospital Pension for the year 1910.

Dr. Collingridge, medical officer of health for the City of London, in his monthly report to the Corporation, mentions that it was found recently that a City firm were advertising as "non-alcoholic cyder" a beverage which did not appear to be genuine cider. Samples were obtained, and they proved to contain 2.6 per cent. of proof spirit and 2.09 grammes per 100 c.c. of cane sugar.

In the House of Commons on February 25 the Chancellor of the Exchequer, in reply to a question if he proposed to introduce legislation during the present session to remove from applicants for old-age pensions the disqualification arising from receipt of Poor Law relief, stated that he hoped to introduce legislation dealing with this matter. In any case the disqualification automatically ceases to operate after the close of the current year.

Dr. C. S. Loch recently gave a lecture on the "Reports of the Royal Commission on the Poor Laws and Relief of Distress," at which he was reported to have said that there were now very few children in the workhouses. It has since been pointed out by Mrs. Barnett that the figures given in the last L.G.B. return, January 1, 1909, were 23,773 for English and Welsh workhouse and infirmary children, a larger number than in any preceding year since 1899.

The Paddington Borough Council at a recent meeting decided to prosecute in cases of failure to notify the medical officer of health under the Notification of Births Act, 1907. Several members protested against the proceedings, as tantamount to persecution, and condemned the Act as an unnecessary interference with the liberty of the subject. It was pointed out by Councillor H. Lidiard that the Council having adopted the statute they were bound to prosecute in cases where it was disregarded.

On the motion for the second reading of the Society of Apothecaries Bill, which appeared on the paper as a private Bill, the Speaker said: It is a Bill which seeks to empower the Society of Apothecaries of London to grant diplomas in sanitary science, public health, and State medicine, and to constitute a School of Hygiene for Tropical Medicine, and also for dentistry and dental surgery, and for that purpose to hold examinations. I have come to the conclusion that this is a Bill which must proceed as a public Bill. The order was discharged and the Bill withdrawn.

In order that those who are about to reside in the tropics, and especially the representatives and employees of commercial and industrial undertakings, may be afforded an opportunity of acquiring a knowledge of the cause and spread of tropical diseases and the means of prevention, the London School of Tropical Medicine has arranged for a course of lectures to be delivered (by kind permission of the West India Committee) at their headquarters in Seething Lane. These lectures are delivered as a sequence to those already given by Professor Simpson at the London Chamber of Commerce. The lectures will be delivered by Dr. C. W. Daniels at 4 p.m. on the 14th, 17th, and 21st inst. Tickets can be had on application to Mr. P. Michelli, Seamen's Hospital, Greenwich, or to Mr. Algernon E. Aspinall, Secretary to the West India Committee, at 15 Seething Lane, E.C.

The Amalgamation of the Savoy Baths.—The hygienic value of bathing is incontestible, and the usefulness of such luxuries as Turkish, Russian, and Electric baths is well known not only to keep individuals healthy, but to help those who are not in good health in certain conditions to regain vitality and energy. In these days when we are living at a high pressure the busy man will do well to consider carefully this question of bathing. The ordinary morning tub is well enough for those who are hardy enough to endure it, but it is not as a hygienic measure to be regarded as equal to a Turkish bath taken once a week or at regular intervals. The absolute necessity, however, in taking a Turkish bath is that the bather shall be comfortable and confident that he is obtaining the best attention under the most sanitary conditions. By the amalgamation of the various Savoy baths, which was celebrated on Tuesday last by a luncheon at the popular Restaurant, given by the manager, Mr. W. Cooper, the London resident is enabled to obtain a most excellent bath, of any kind or variety he fancies, among surroundings that recall to mind the luxury prevailing at the private baths in Vienna. The following are the institutions under Mr. Cooper's management: Ladies and gentlemen (open daily), 24 Railway Approach, London Bridge, London, S.E.; 459 and 461 Brixton Road, London, S.W. Ladies only (open daily), 12 York Street, Jermyn Street, Piccadilly, London, S.W.; Denman Street, London Bridge, London, S.E. Gentlemen only (open daily), Savoy Mansions, Savoy Street, Strand, London, W.C.; 9 Caledonian Road, King's Cross, London, N. Night and day baths (never closed), 91 and 92 Jermyn Street, Piccadilly, London, S.W. Savoy hydro (residential accommodation), 459 Brixton Road, London, S.W. The charges are moderate and the attendance is exceptionally good, so that these establishments may be confidently recommended. The management issues an attractive illustrated pamphlet, "A Guide to Health and Hygiene," which contains much useful information.

